# Neuropsychological functioning in inpatients with major depression or schizophrenia

**DOI:** 10.1186/1471-244X-13-203

**Published:** 2013-08-02

**Authors:** Annette Schaub, Nicole Neubauer, Kim T Mueser, Rolf Engel, Hans-Jürgen Möller

**Affiliations:** 1 Department of Psychiatry and Psychotherapy, University of Munich, Nußbaumstr 7, D-80336, Munich, Germany; 2 Psychological Psychotherapy, Rheinstr 30, 80803, Munich, Germany; 3 Center for Psychiatric Rehabilitation, Boston University, 940 Commonwealth Avenue West, Boston, MA 02215, USA

**Keywords:** Neuropsychological functioning, Depressive disorder, Schizophrenia, Psychopathology, Post-acute stage of the illness

## Abstract

**Background:**

Studies that compare neuropsychological functioning in inpatients with mood disorder or schizophrenia come to heterogeneous results. This study aims at investigating the question whether there are different neuropsychological test profiles in stabilised post-acute inpatients with affective disorders or schizophrenia.

**Method:**

We were interested in evaluating impairment in specific areas of cognitive functioning in patients with schizophrenia or depression. In clinical reality, patients with depression and schizophrenia are often treated together with little attention to their specific needs. 74 patients with major depression and 38 patients with schizophrenia were assessed in a comprehensive neuropsychological battery. All patients were in a post-acute stage of their illness, i.e. remission of acute symptoms.

**Results:**

In spite of a comparable mean score of psychopathological symptoms in the Brief Psychiatric Rating Scale-Expanded (BPRS-E) as well as in the Global Assessment Functioning Scale (GAF), patients with depressive disorder showed significantly better results in verbal and visual short-term memory, verbal fluency, visual-motor coordination, information processing in visual-verbal functioning and selective attention compared to patients with schizophrenia. No significant differences between both samples were found in practical reasoning, general verbal abstraction, spatial-figural functioning, speed of cognitive processing.

**Conclusions:**

These results show that there are differences in scores in psychopathology (BPRS-E, GAF) in patients with affective disorders or schizophrenia and different neuropsychological test profiles in the post-acute stage of their illness.

## Background

Impaired neuropsychological functioning in schizophrenia has been evaluated in range of meta-analyses focusing on schizophrenia (Heinrichs and Zakzanis [1], schizophrenia or bipolar disorder (Krabbendam et al. [2], and first-episode schizophrenia (Mesholam-Gately et al. [3]. Heinrichs and Zakzanis's [1] seminal meta-analysis of predominantly chronic schizophrenia samples showed moderate to large effect sizes (d > .60) for all 22 neurocognitive test variables. The results indicated that schizophrenia is characterized by broad-based cognitive impairment, with varying degrees of deficit across all ability domains measured by standard clinical tests. Krabbendam et al. [2] analysed 31 studies and demonstrated that patients with bipolar disorder show better cognitive performance than schizophrenia. Mesholam-Gately et al. [3] focused their meta-analysis on 47 studies of first-episode schizophrenia. They found that impairments are reliably present across the range of cognitive domains, and are comparable to the degree of impairment present in patients with a well-established illness; the most pronounced impairments were in immediate verbal memory and processing speed.

The considerable heterogeneity of effect sizes across studies, however, underscores the variability in the impact of the illness on cognitive functioning. Studies of neuropsychological functioning comparing acutely depressed patients to those with schizophrenia show similarly heterogeneous results (Albus et al. [4]. Patients with depression may be expected to fall between normal and schizophrenia groups, with considerable overlap with normal, and unknown and/or controversial overlap with schizophrenia.

Albus et al. [4] compared patients with a first episode of either schizophrenia or major depression and found that depressed patients performed better in visual-motor and attentional functioning. Mood disorder patients without psychotic features did not perform significantly differently from healthy normal controls, although those with psychotic features performed as poorly as those with schizophrenia. Albus et al. [5] reassessed the same patients with first-episode schizophrenia, chronic schizophrenia, and healthy controls again after five years and concluded that neuropsychological impairment had already been present at the onset of the illness and remained stable over the early course of schizophrenia. When controlling for relevant confounding variables, neuroleptics showed a deleterious influence only on verbal fluency, but no other measures of neuropsychological performance. Table 1 provides an overview of studies related to neuropsychological differences between schizophrenia and depression.

**Table 1:** Overview of studies related to neuropsychological differences in schizophrenia or depression

**Author**	**Franke et al. (1993)**[6]	**Albus et al. (1996)**[4]	**Mitrushina et al. (1996)**[7]	**Verdoux and Liraud (2000)**[8]	**Egeland et al. (2003)**[9]	**Reichenberg et al. (2008)**[10]
Patients	30 patients with schizophrenia (S) 15 unipolar non-psychotic patients (D) 30 control persons (Cont)	27 patients with schizophrenia (S) 10 depressed patients (D), 17 patients with bipolar disorder (B) = 27 patients with affective disorder, 27 control persons (Cont). All patients were first episode.	25 depressed patients. (D), 17 patients with mania (M), 21 patients with schizophrenia (S), 18 patients with schizoaffective disorder (SA), 22 patients with other psychosis (P) All patients had psychotic symptoms.	20 patients with schizophrenia (S), 29 patients with other psychosis (P), 33 patients with bipolar disorder (B), 19 patiens with Major Depression (D)	53 patients with schizophrenia (S), 50 patients with recurrent depression (D), 50 healthy controls (Cont)	94 patients with schizophrenia (S), 15 patients with schizoaffective disorder (SA), 78 patients with bipolar disorder (B), 48 patients with major depression (D)
Neuropsychological Test Battery	Wisconsin Card Sorting test (WCST), Verbal Fluency Test (VFT), Trail Making Test (TMT A and B), Digit Span Test (DST)	Wechsler Adult Intelligence Scale (WAIS-R), WCST, Verbal fluency Test, Wechsler Memory Scale (WMS-R), California Verbal Learning Test, TMT, Stroop Test, Continous Performance Test, Span of Apprehension Test	Neurobehavioral Cognitive Status Examination (NCSE; Kiernan et al. 1987)	BEM-84 (Memory), WCST, STROOP	Continous Performance Test, dichotomic listening, STROOP, 2 Visual-motor coordination speed	WAIS-R, WMS-R, STROOP, TMT, Finger Tapping Test, Facial Recognition Test, Letter Fluency and Sentence Repetition Test
Results	WCST, TMT, VFT: S,D < Cont TMT B: S < D	Visual-motor coordination: S < A A without psychotic symptoms = Cont A with psychotic symptoms = S	Memory, abstract thinking: distinct deficits in patients with schizophrenia (S < D)	Global memory functioning: S < D; verbal memory: S < P, B, D; WCST, STROOP: no differences	Speed and selective attention: S < Cont Speed: D < Cont Vigilance: D < S	S < SA < B < D

< = “worse than”.

*S* Schizophrenia, *D* Major depression, *Cont* healthy control group, *B* bipolar disorder, *P* other psychosis, *AffectD* affective disorder.

Franke et al. [6] compared neuropsychological functioning in acute patients with schizophrenia or nonpsychotic major depression, who were not currently receiving medication, to healthy controls. They found only small differences in executive functions, verbal fluency, and focused attention between patients with depression or schizophrenia. Mitrushina’s study [7] showed that patients with an acute psychotic episode had significantly lower scores in memory functioning and in reasoning than patients with depression. Verdoux and Liraud [8] also found that patients with schizophrenia had poorer memory functioning than those with depression, although there were no differences in executive functioning performance. Egeland et al. [9] found that depressed patients showed significantly better performance in working and declarative memory than schizophrenia patients. Reichenberg et al. [10] investigated neuropsychological functioning in schizophrenia and mood disorder two years after index hospital admission. Both groups had impairments in memory, executive functioning, attention and processing speed; however, patients with schizophrenia were more impaired across all cognitive domains. Impairment in neuropsychological performance is common in people with major depression with psychotic features. However, as in schizophrenia, a minority of these individuals perform within the normal range, with estimates of the prevalence of normal psychological functioning ranging between 42% and 77% in major depression, compared to 16% to 45% in schizophrenia.

This study investigated whether there were significant neuropsychological differences between post-acute inpatients with major depression compared to those with schizophrenia. In addition, the relationships between symptoms and neurocognitive performance were evaluated.

## Methods

### Setting and sample

The study was conducted with inpatients in the Department of Psychiatry and Psychotherapy, Ludwig Maximilian University of Munich. After receiving permission of the ethical committee, all potentially eligible patients were approached to participate in the study. A total of 112 patients provided signed informed consent and were included in the study, which began in February 1998 and ended in November 2000. Inclusion criteria were: ICD-10 diagnosis of schizophrenia or major depression; hospitalized for treatment of an acute episode of the illness; and sufficient symptom stabilization to permit participation in the neuropsychological evaluation. All patients were treated with psychopharmacological in-terventions.

Thirty-eight patients had schizophrenia (17 women, 21 men) and 74 had major depression (38 women, 36 men), a similar gender distribution for these diagnoses to that seen in this hospital. Table 2 summarizes the diagnoses of the sample and Table 3 describes their demographic and clinical characteristics.

**Table 2:** Distribution of diagnosis (ICD-10) post-acute inpatients with depression (n = 74) or schizophrenia (n = 38)

**Depressive disorder**	
Moderate or minor depression	1 (1.4%)
Remitted	1 (1.4%)
Minor depressive episode	2 (2.7%)
Moderate depressive episode	14 (19%)
Severe depressive episode without psychotic symptoms	19 (25.7%)
Severe depressive episode with psychotic symptoms	6 (8.1%)
Moderate episode	6 (8.1%)
Moderate episode without psychotic symptoms	10 (13.5%)
Severe depressive episode with psychotic symptoms	
within a recurrent disorder	3 (4.1%)
unknown	12 (16.2%)
**Schizophrenia**	
Paranoid schizophrenia	21 (55.3%)
Hebephrene schizophrenia	3 (7.9%)
Catatone schizophrenia	1 (2.6%)
Undifferentiated schizophrenia	3 (7.9%)
Schizophrenia simplex	1 (2.6%)
Delusional disorder	2 (5.3%)
Acute polymorphe psychotic disorder	3 (7.9%)
Acute polymorphe psychotic disorder with schizophrenia	1 (2.6%)
Acute schizophreniform psychotic disorder	2 (5.3%)
Schizophrenic disorder at present manic	1 (2.6%)

**Table 3:** Chi-square tests comparing patients with major depression (n = 74) and schizophrenia (n = 38) on categorical demographic characteristics and hospitalization status

**Variables:**	**Major depression**	**Schizophrenia**	**Chi-Square**	**df**	**Signif.**
Sex:					
Women	38 (51.4%)	17 (44.7%)	.440	1	.507
Men	36 (48.6%)	21 (55.3%)			
Marital status:					
Never married	22 (31.4%)	24 (63.2%)	10.14	1	**.001**
Ever married	48 (68.6%)	14 (36.8%)			
Education:					
Elementary education	26 (37.1%)	18 (47.4%)	1.07	1	.302
Higher education	44 (62.9%)	20 (52.6%)			
Work qualification:					
Unskilled	16 (23.2%)	13 (34.2%)	1.51	1	.220
Skilled worker	53 (76.8%)	25 (65.8%)			
Employment status:					
Unemployed	26 (37.1%)	17 (44.7%)	.593	1	.441
Employed	44 (62.9%)	21 (55.3%)			
Disorder:					
First admission	50 (72.5%)	36 (94.7%)	7.71	1	**.006**
Multiple admission	19 (27.5%)	2 (5.3%)			

*The boldface data are to mark the significance of the data: p*<05; p**<0.01; p***<0.001.

### Instruments

Diagnoses were made according to ICD-10 criteria and drawn from charts. Symptoms were assessed with the Brief Psychiatric Rating Scale Expanded (BPRS-E [11]), the Global Assessment of Functioning Scale (GAF, [12], the Scale for the Assessment of Negative Symptoms (SANS [13], Munich version [14], the Hamilton Depression Scale (HAMD, [15], and the Montgomery Asberg Depression Rating Scale (MADRS, [16]. The factors for the BPRS-E were based on those from Velligan’s [17] factor analysis, including: depression/anxiety, activation, retardation and psychosis.

All participants were also evaluated with a comprehensive neuropsychological battery. Consistent with previous studies of neuropsychological functioning in schizophrenia and mood disorders [1-11] the individual measures administered to all subjects were selected to assess verbal intelligence, fluency, visual-figural problem solving, simple and focused attention as well as verbal learning and memory. The instruments included the German Version of the Auditory Verbal Learning Memory Test (VLMT, Helmstedter et al. [18], Word fluency Test from Regensburg (RWT; Aschenbrenner et al. [19], Visual test I and II from the Wechsler Scale [20], Subscales of the German Wechsler Adult Test Revised (WAIS-R) [21]: Information, Similarities, Picture Completion, Block Design, Digit symbol test and the German version of the Colour Word Interference test (STROOP; Bäumler [22].

A subset of the overall assessment measures was selected for analysis in this study based on the availability of comprehensive norms, including VLMT, RWT, Visual test, WAIS-R and STROOP. The symptom assessments and neuropsychological tests were administered by psychologists with experience in evaluating patients with severe mental illness.

### Data analysis

Chi-square tests were conducted to compare patients with major depression and schizophrenia on categorical demographic variables and history of hospitalization (first episode vs. multiple episode). Analyses of variance (ANOVAS) were performed to compare the two groups on continuous demographic variables and the symptom measures. The patients with major depression were significantly older than those with schizophrenia. To statistically adjust for the potential effects of age on the comparisons of the groups on the cognitive measures, analyses of covariance (ANCOVAS) were performed, including age as a covariate.

There are over 30 dependent variables and therefore we did a (Bonferonni) correction for multiple analyses (.05/30 = .00167). Therefore, effects significant at p < .002 were considered statistically significant, whereas effects significant a the conventional p < .05 but not the Bonferroni corrected level were considered trends.

## Results

### Demographic and clinical data

In addition to being older, the patients with depression and schizophrenia differed significantly in marital status and history of prior hospitalisations. Depressed patients were more likely to have been married and to have had prior hospitalizations than patients with schizophrenia. There were no significant differences in age at onset, duration of illness, education, total duration of inpatient treatment or total scores in BPRS-E and in GAF. However, there were significant differences in several of the BPRS-E subscales. Depressed patients had significantly worse scores on the subscales depression, anxiety, suicidality and hostility, and significantly less severe scores on the psychosis subscales such as suspiciousness, hallucinations, unusual thought, bizarre behaviour, grandiosity and conceptual disorganisation than patients with schizophrenia. Table 4 summarizes the results.

**Table 4:** One-way ANOVAS focusing on continuous demographic variables and symptoms (F2-Diagnosis: n = 38, F3-Diagnosis: n = 74)

**Variable**	**Diagnoses**	**M**	**SD**	**df**	**F**	**Significance**
Age	F2	33.50	12.14	(1,110)	9.826	**.002**
	F3	41.07	12.08			
Age at onset	F2	29.58	11.71	(1,106)	3.786	.054
	F3	34.47	12.87			
Duration of illness	F2	4.17	7.10	(1,106)	2.199	.141
	F3		6.61	8.70		
Number of Hospitalisations	F2	1.05	0.23	(1,105)	5.666	**.019**
	F3	1.67	1.58			
Total length of hospitalisations	F2	5.32	2.11	(1,105)	2.885	.092
	F3	8.16	10.18			
GAF	F2	55.08	12.86	(1,101)	.234	.630
	F3	53.95	10.33			
BPRS	F2	37.00	6.82	(1,100)	.268	.606
	F3	37.75	7.22			
Anxiety	F2	2.18	1.04	(1,100)	15.24	.000
	F3	3.19	1.37			
Depression	F2	1.68	.96	(1,100)	32.43	.000
	F3	3.14	1.39			
Suicidality	F2	1.16	.72	(1,100)	6.05	.016
	F3	1.64	1.07			
Hostility	F2	1.05	.32	(1,100)	12.83	.001
	F3	1.39	.79			
Grandiosity	F2	1.39	1.00	(1,100)	6.88	.010
	F3	1.05	.28			
Suspiciousness	F2	2.21	1.09	(1,100)	12.83	.001
	F3	1.56	.73			
Hallucinations	F2	1.58	1.73	(1,100)	5.62	.020
	F3	1.05	.38			
Unusual thought	F2	1.63	1.08	(1,100)	7.72	.000
	F3	1.06	.30			
Bizarre behaviour	F2	1.89	1.39	(1,100)	20.31	.000
	F3	1.06	.39			
Conceptual disorganization	F2	1.26	.60	(1,100)	4.15	.044
	F3	1.06	.39			
SANS(F2)	F2	29.89	20.61			
Single score						
SANS (F2)	F2	6.97	4.75			
Total score						
MADRS (F3)	F3	19.04	7.85			
HAMD (F3)	F3	17.28	6.99			

*The boldface data are to mark the significance of the data: p*<05; p**<0.01; p***<0.001.

### Neuropsychological functioning

The results of the ANCOVAs comparing cognitive performance in the patients with depression with schizophrenia are summarized in Table 5. Patients with depression had significantly better cognitive performance than those with schizophrenia on the following variables: VLMT; immediate recall of words, verbal learning, free recall, both subtests of verbal fluency (RWT) (letters and; categories); the Visual test and Digit symbol coding on the HAWIE, as well as two sub-items of STROOP. The remaining variables showed no significant differences between the two samples in the post-acute stage of illness.

**Table 5:** Oneway ANCOVAS focusing on neuropsychological tests (F2-Diagnosis: n = 38, F3-Diagnosis: n = 74) with age as covariate

**Variable**	**Diagnoses**	**M**	**SD**	**F**	**Significance**
VLMT	F2	5.68	1.96	11.20	**.001**
Immediate memory	F3	6.58	1.88		
VLMT	F2	47.42	10.80	8.83	**.004**
Learning (1–5)	F3	50.16	10.41		
VLMT	F2	9.21	3.27	8.71	**.004**
Free recall	F3	10.27	3.69		
VLMT	F2	.9442	.0611	.045	.832
Embedded recall	F3	.9277	.072		
RWT	F2	43.71	9.88	21.75	**.000**
Categories	F3	53.45	12.53		
RWT	F2	36.29	15.83	8.65	**.004**
Letter	F3	43.43	14.16		
Visualtest	F2	64.37	32.75	2.66	.106
Immediate	F3	70.88	29.81		
Visualtest	F2	54.76	34.94	4.64	**.033**
Short-term	F3	64.18	34.27		
HAWIE	F2	11.51	3.45	.388	.535
Information	F3	11.69	3.33		
HAWIE	F2	11.78	3.74	1.44	.223
Similarities	F3	12.48	3.39		
HAWIE	F2	9.61	4.45	2.77	.100
Picture completion	F3	10.55	4.17		
HAWIE	F2	9.31	4.30	1.45	.232
Block design	F3	9.84	3.18		
HAWIE	F2	8.14	2.66	12.16	**.001**
Digit symbol test	F3	10.18	3.18		
STROOP: fwl	F2	44.76	7.77	16.63	**.000**
STROOP: fwl	F3	53.17	8.38		
STROOP: fsb	F2	47.32	7.75	18.99	**.000**
STROOP: fsb	F3	51.99	9.37		
STROOP: T-Score	F2	47.86	11.21	.575	.568
NOM	F3	49.41	9.12		
STROOP: T-Score	F2	52.49	8.46	.000	.991
SEL	F3	52.34	9.25		
STROOP: Age-related	F2	45.24	7.93	11.68	**.001**
norm fwl	F3	51.62	7.93		
STROOP: Age related	F2	45.08	8.45	12.73	**.001**
norm fsb	F3	52.18	9.49		
STROOP: Age related-	F2	47.03	8.68	12.30	**.001**
norm int	F3	53.09	7.08		

*The boldface data are to mark the significance of the data: p*<05; p**<0.01; p***<0.001.

## Discussion

### Illness related differences

The results of this study found differences in neuropsychological functioning between patients with depression and patients with schizophrenia in the post-acute stage of their illness. Similar differences have been reported in several previous studies. In spite of comparable total scores in BPRS-R and GAF, patients with post-acute depression show significantly better results in several cognitive assessments (verbal memory and fluency, visual-motor concentration, information processing in visual-verbal areas and selective attention) compared to their counterparts. Special attention should be paid to the following result: Patients with depression were significantly older and had a significantly higher number of hospitalizations. These aspects might indicate a more severe course of the illness and hint at worse performing in neuropsychological functioning. However, there were no significant differences in other cognitive variables such as verbal comprehensiveness, reasoning, spatial perception and visual abstract processing, cognitive processing, and abstract cognitive reasoning.

### Comparison to other studies

Comparing the results of this study with other studies the following conclusions can be drawn. Many studies [3-10] show better performance in patients with depression than in patients with schizophrenia. Comparing our study with the results of Reichenberg [10] shows similarities to Albus et al. [3]: patients with schizophrenia show the lowest level of neuropsychological functioning compared to depression and bipolar disorder, however, psychotic features turn out to be the most important characteristics.

### Strengths and limitations

Shortcomings are due to different sample sizes. There was no matching of the patients according to age and duration of illness to control for demographic and clinical variables and their impact on the results. It should be pointed out that this study did not include a control group of non-psychiatric patients or healthy individuals, however, the overall assessments measures were selected for analysis in this study because of the availability of comprehensive norms. The large majority of the patients were under pharmacological treatment. In comparison to other studies we found that the treatment with neuroleptics [23] improved cognitive functioning, however, there is also some evidence that drugs with anticholinergic properties may have a negative impact on memory performance [24].

We analysed several sociodemographic and clinical attributes, however, there were only significant differerences with regard to marital status, first admission versus multiple admission recurrent, number of hospitalisations and especially age. Future studies are to investigate whether the given neuropsychological differences between patients with acute schizophrenia or depression continue to persist in the course of the illness i.e. patients with different disorders recover differently from their acute episode. The non-significant difference in the BPRS-E total score between major depression and schizophrenia does not reveal information about the strength of the symptom cluster. The same total score may reveal a different profile of symptoms. We agree to the consensus that schizophrenia is marked by cognitive impairments that do not appear to be secondary to symptoms and point to localized brain dysfunction. The addition of functional competence to standard cognitive test data [25] yields a significant increase in validity only for concurrent and not for longitudinal prediction of community independence. The specific real-world validity of functional competence is modest and this information is largely redundant with standard cognitive performance.

### Implications of findings in aiding patient management

During the last 20 years illness management programmes gained importance in the treatment of schizophrenia. Illness management is a broad set of strategies designed to help individuals with serious mental illness collaborate with professionals in managing their mental illness, reduce their susceptibility to the illness (e.g., relapses, effects on functioning), cope with their symptoms, and discover (or rediscover) their strengths and abilities for pursuing personal goals (Mueser et al. [26]. These programmes are based on the vulnerability stress model [27] and its further development enriched with coping and competency [28] as well as the transtheoretical model [29] that proposes motivation to change over a series of stages asking for motivational interviewing at the earliest stage to help clients identify and pursue their personal goals and to explore how improved illness management can help them achieve these goals.

At the Department of Psychiatry and Psychotherapy, Ludwig Maximilian University of Munich, illness management programmes were set up since 1995 for patients with schizophrenia [30-32] or mood disorder [33,34] including groups for patients as well as distinct groups for relatives adapted to the needs of acute psychiatry. The programmes include psychoeducational elements (e.g. handouts for important topics) and cognitive behavioural learning principles including building up rewarding activities, stress management skills, cognitive restructuring and relapse prevention. These groups are based on sound didactic and behavioural principles as patients in severe psychiatric illness suffer from deficits in neuropsychological functioning that have to be compensated [31,32]. Strategies include personalization of treatment, rewarding and positive social interactions, illustration and summarizing important information, as well as acknowledging overstimulation (e.g. providing a “chair to rest” without questions being asked for overloaded patients, however, rewarding the patient’s presence). Differences in neuropsychological functioning between these two samples led to a shorter duration and less demanding cognitive strategies and homework assignments in the programme for schizophrenia compared to those in mood disorder.

Cognitive therapy has recently gained prominence in the treatment of schizophrenia with process and content interventions being two general approaches to address cognitive dysfunctions. Whereas process interventions aim at remediating basic information-processing skills that serve as vulnerability markers for further episodes (e.g. Wykes and Spaulding [35] content approaches (e.g., Beck et al. [36] focus on changing the nature of or one’s response to dysfunctional thoughts and, or unlike the latter put more emphasis on stress management (e.g., Schaub et al. [30], however relatively few patient characteristics were predictive of benefit from participations in these programmes [32]. In adapting CT for low-functioning patients with schizophrenia, Beck and his group [36] shifted the emphasis from the predominantly symptom-oriented approach to a person-oriented therapeutic approach by highlighting the patients’ interests, assets, and strengths. The objective is to improve the level of functioning in the form of enhanced productivity, independence, and quantity and quality of social interactions. Beck et al. [37] developed the framework for this therapy from the finding that dysfunctional beliefs, in conjunction with neurocognitive impairment, impede functioning.

Another treatment approach is largely influenced by the principles and spirit of the Recovery Movement (Mueser et al. [26]). In a group of patients with cognitive deficits this approach turned out to be efficient and therefore seems promising for this group of patients as a low level of cognitive functioning may turn out as a rate limiting factor for therapy Mueser et al. [38]. The work on CBT and so-called cognitive remediation is broad and diverse, covers a range of paradigms relevant to the findings of this study, and is beginning to reveal complex relationships between baseline functioning and treatment effects. In fact, the neuropsychological level of analysis of this study refers less to the schematic approach of Beck than to most other CBT approaches for psychosis. The overview of Wykes and Spaulding [35] as well as the study by Silverstein et al. [39] are promising in so far as attention shaping e.g. turned out to be an effective example of support cognition, in that cognitive abilities are improved within the environmental context where the patient is experiencing difficulty, leading to gains in both attention and functional outcome.

This study refers to post- acute inpatients with low symptom profiles that were close to remission. The low symptom profile limits generalization as due to the new health system with shorter inpatient stay patients are less likely to be in such a stable state. Patients with schizophrenia showed lower cognitive functioning compared to patients with mood disorder, however, they could also benefit from illness management programmes if these were to compensate the patients’ cognitive deficits.

## Conclusions

In summary we found that in spite of comparable total scores in psychopathology (BPRS-E, GAF) patients with affective disorders compared to patients with schizophrenia showed different neuropsychological test profiles in the post- acute phase of their illness. Schizophrenia is marked by cognitive impairments that do not appear secondary to symptoms.

The different results in neurofunctioning in mood disorder and schizophrenia laid the foundation for different illness management programmes in both samples that re to compensate for neuropsychological deficits.

## Competing interests

All authors declare that they have no competing interest.

## Authors’ contributions

AS, designed and interpreted the original study. NN analyzed the data. KM, RE and H-JM also have contributed to and have approved the final manuscript. All authors read and approved the final manuscript.

## Pre-publication history

The pre-publication history for this paper can be accessed here:

http://www.biomedcentral.com/1471-244X/13/203/prepub
